# Natural Bioactive Compounds in Rheumatoid Arthritis: Experimental Evidence from Adjuvant Arthritis Model Supporting Combination Strategies with Methotrexate

**DOI:** 10.3390/ijms27177968

**Published:** 2026-09-07

**Authors:** Mohammad Umar, Waqar Ahmad, Katarina Bauerova

**Affiliations:** 1 Centre of Experimental Medicine, Institute of Experimental Pharmacology and Toxicology, Slovak Academy of Sciences, 841 04 Bratislava, Slovakia; umar.mohammad@savba.sk (M.U.); ahmad.waqar@savba.sk (W.A.); 2 Faculty of Natural Sciences, Comenius University in Bratislava, 842 15 Bratislava, Slovakia

**Keywords:** rheumatoid arthritis, adjuvant arthritis, natural bioactive extracts and compounds, methotrexate combination therapy, oxidative stress, NF-κB signaling, JAK-STAT pathway, Nrf2 antioxidant pathway

## Abstract

Rheumatoid arthritis (RA) is a chronic autoimmune inflammatory disease that represents continuous synovial inflammation, oxidative stress, immune dysregulation, and progressive cartilage degradation and bone erosion. Although disease-modifying antirheumatic drugs (DMARDs), including methotrexate (MTX), have improved clinical outcomes, treatment-limiting adverse effects remain a concern. Increasing evidence suggests that natural bioactive compounds may serve as adjunctive approaches by modulating multiple pathogenic pathways. This review summarizes biomarker-based modulation of inflammatory, oxidative, and immune pathways by plant-derived extracts, nutraceuticals, and biologically derived compounds, with particular emphasis on evidence from adjuvant arthritis (AA). AA is a widely used experimental model that reproduces several inflammatory and oxidative features relevant to RA, including cytokine activation, NF-κB/MAPK signaling, Th17/JAK-STAT3 signaling, redox imbalance, and tissue degeneration. Studies in AA indicate that selected natural compounds, alone or in combination with MTX, can reduce inflammatory cytokines (e.g., IL-1β, IL-6, IL-17A), matrix-remodeling markers (e.g., MMP-9), and oxidative-stress markers (e.g., protein carbonyls and lipid peroxidation) while supporting antioxidant defenses (e.g., HO-1 and CAT). Overall, natural bioactive substances may have potential as adjunctive candidates for further investigation in RA, particularly because of their effects on inflammatory and oxidative pathways. Preclinical studies of MTX combinations have reported additional improvements in selected disease-associated outcomes; however, these findings require confirmation in well-designed clinical studies. This review critically evaluates the preclinical evidence for natural bioactive compounds, with a focus on mechanistic studies in AA and the potential for combination strategies with methotrexate.

## 1. Introduction

The most prevalent progressive and systemic autoimmune disease, rheumatoid arthritis (RA), is characterized by a persistent inflammatory process that primarily affects the lining of the synovial joints (articular damage), with gradual cartilage degradation and bone erosion, as well as affecting other organs and body systems, including blood vessels [1,2,3]. As the disease progresses, joint tissues become severely injured and deformed, thereby leading to systemic and extra-articular problems, such as infection, lymphoma, osteoporosis, cardiovascular disease, neurological disorders, and pulmonary and metabolic problems [4,5]. Despite therapeutic advancements, the etiology of RA is still poorly understood, and there is currently no recognized cure [6].

The pathogenesis of RA is complex and multifactorial, involving immune response malfunction, pro-inflammatory cytokine production increases, excess oxidative and nitrosative stress, and altered activation of intracellular signaling pathways. Key inflammatory mediators such as tumor necrosis factor-α (TNF-α), interleukins (IL-1β, IL-6, and IL-17), as well as prostaglandins, drive synovial hyperplasia and pannus formation, while matrix metalloproteinases contribute to cartilage and bone destruction. These processes are regulated by several pathways, including nuclear transcription factor-κB (NF-κB) and mitogen-activated protein kinase (MAPK) pathways, and are tightly regulated by intracellular signaling cascades [7,8]. Together, these pathways support both degenerative and inflammatory processes in RA. Among them, one of the most common dysregulated pathways in RA is the MAPK pathway. Further, a major factor in the development of RA and many other autoimmune disorders is the stimulation of NF-κB-dependent gene expression. NF-κB is a family of structurally connected and evolutionarily conserved proteins (NF-κB2, NF-κB1, and p65,) that work as homodimers or heterodimers and control the expression of many genes involved in RA, including TNF, IL-1, IL-6, cyclo-oxygenase-2, chemokines, inducible nitric oxide synthases, and matrix metalloproteinases (MMPs). Furthermore, TNF and IL-1 are powerful NF-κB activators in and of themselves [9,10].

In parallel, oxidative and nitrosative stress play fundamental roles in the initiation and progression of RA. There has also been evidence of a mutual relationship between oxidative stress and inflammation in RA. Inflammation occurs due to oxidative stress, and increased levels of inflammation also increase oxidative stress. This process ultimately leads to synovitis, a key pathological process for RA [11]. Reactive oxygen species (ROS) are constantly produced in normal physiology. However, when ROS are overproduced and antioxidant defence systems are dysfunctional, oxidative stress is triggered. Such imbalance may lead to the loss of essential macromolecules and cells. This may interfere with the normal physiological processes of the whole organism. Furthermore, oxidative stress results in lipid peroxidation, protein oxidation and redox-sensitive inflammatory pathways, establishing a vicious circle that enhances oxidative damage and inflammation. This redox imbalance is of key pathophysiological significance in RA. Clinical and experimental evidence suggests alterations in antioxidant levels and a decline in antioxidant defence systems [12,13].

The treatment for RA mainly involves disease-modifying antirheumatic drugs (DMARDs), and among these, methotrexate (MTX) is still considered the cornerstone for treatment. MTX shows anti-inflammatory effects by inhibiting folate metabolism pathways, reducing the proliferation of immune cells, and regulating cytokine expression. In synovial inflammation, the anti-inflammatory effects of the MTX stem has the capacity to inhibit cellular proliferation by decreasing purine and pyrimidine synthesis. T lymphocytes are mostly affected by this process. MTX is known to be effective in the treatment of malignant diseases, but constant use of methotrexate is often linked to various side effects, like hepatotoxicity, gastrointestinal sensitivity, hematological abnormalities, and oxidative stress-induced tissue damage. Furthermore, the incomplete response of most patients to treatment, which delays achieving continuous clinical remission, supports the need for novel treatment approaches [14,15,16].

In this context, experimental RA models have been valuable in clarifying disease mechanisms and treatment approaches. Among these, adjuvant arthritis (AA) is one of the most widely used experimental models. AA can be induced by complete Freund’s adjuvant in susceptible rodent strains, including Lewis rats. The model reproduces several features relevant to human RA, such as synovial inflammation, pannus formation, cartilage damage, bone erosion, systemic inflammation, and oxidative-stress-associated tissue injury. Natural biological extracts and compounds have therefore been investigated in AA as monotherapies and in combination with MTX. However, even though adjuvant-induced arthritis (AA) is a well-established and reproducible model for studying inflammatory and immune-mediated mechanisms relevant to rheumatoid arthritis (RA), it does not fully recapitulate the pathogenesis, chronicity, or immunological heterogeneity of human disease. Moreover, despite the involvement of adaptive immune responses, the presence and contribution of rheumatoid factors (RFs), anti-citrullinated protein antibodies (ACPAs), and other autoantibodies may vary between experimental models and species, limiting the representation of autoantibody-associated heterogeneity of human RA. The mechanisms observed in this model may also be influenced by the exogenous adjuvant that is utilized to induce AA, which may also affect the inflammatory pathways activated. Besides that, interspecies and strain-specific differences in immune modulation, drug metabolism, transport, and pharmacokinetics may influence both disease phenotype and therapeutic response. Another limitation is dose translation, as identical mg/kg doses may not result in similar systemic exposure in rodent species and humans, especially with respect to natural substances with poor bioavailability. Accordingly, findings from AA are best interpreted as mechanistic and preclinical evidence that may inform translational research rather than as direct evidence of clinical efficacy [8,17,18,19,20,21].

Recently published systematic reviews and meta-analyses have added weight to clinical evidence in support of the therapeutic potential of natural bioactive compounds in RA. Qiuwei Peng et al. conducted a systematic review and network meta-analysis of 18 randomized controlled trials (RCTs) that tested the efficacy of natural products derived from plants. Among them, curcumin produced a positive effect on inflammatory markers and swollen and tender joints; quercetin on pain; and resveratrol on disease activity scores [22]. Likewise, in another meta-analysis of 47 RCTs, dietary polyphenols, including curcumin, resveratrol, quercetin, hesperidin, ginger, pomegranate, and saffron, were shown to be effective in improving disease activity, inflammatory markers, and some oxidative stress markers [23]. The possible beneficial effects of certain phytotherapeutic interventions, including *Curcuma longa*, *Zingiber officinale*, *Tripterygium wilfordi*, and *Salvia miltiorrhiza*, have been observed in RA therapy [24]. However, the high heterogeneity with respect to compounds, their preparation, dosages, duration of therapy, associated treatments, and measured outcome indices, as well as insufficient evidence on individual compounds, provides limited information on the molecular mechanisms underlying these effects. Moreover, clinical research has not yet fully clarified the pathway-specific processes and related biomarker alterations [25]. The earlier experimental overview by Bauerová et al. primarily summarized experimental findings on natural substances and their potential relevance to arthritis therapy [26]. The present review adopts a focused mechanistic and translational framework. It specifically integrates biomarker-based evidence from AA models with the evaluation of natural bioactive compounds as potential adjuncts to MTX while critically considering clinical evidence, pharmacokinetic interactions, standardization, and limitations affecting translation to RA therapy.

## 2. Materials and Methods

A structured narrative review was conducted to evaluate evidence for naturally occurring bioactive chemicals, with a focus on their molecular mechanisms, effects in experimental models of arthritis, and their potential for use alone or in combination with methotrexate (MTX) in RA. Original experimental studies with specific mechanistic evidence and relevant clinical studies were prioritized, and current reviews were included for translational perspectives. This approach allows the integration of knowledge on inflammatory and immunological signaling, oxidative stress and antioxidant defenses, and cartilage and bone degeneration.

### 2.1. Search Strategy

A literature search was performed in PubMed, Google Scholar, Scopus, and Web of Science databases. For the first exploratory search, we did not impose a strict date filter on publications. We updated the search during the rewriting of this manuscript to find new research and to check the mechanistic evidence presented in this review.

The search strategy consisted of combinations of the following keywords: “rheumatoid arthritis”, “adjuvant arthritis”, “natural compounds”, “bioactive compounds”, “plant extracts”, “phytochemicals”, “methotrexate”, “oxidative stress”, “reactive oxygen species”, “inflammation”, “Nuclear factor kappa-light-chain-enhancer of activated B cells (NF-κB)”, “mitogen-activated protein kinase (MAPK)”, “Janus kinase-signal transducer and activator of transcription (JAK/STAT)”, “phosphoinositide 3-kinase/protein kinase B (PI3K/Akt)”, “Nuclear factor erythroid 2-related factor 2 (Nrf2)”, “Heme oxygenase-1 (HO-1)” and “matrix metalloproteinases (MMPs)”. Boolean operators (AND/OR) were used to link the disease, intervention, and mechanism-related terms. Further searches were conducted for natural ingredients and bioactive compounds mentioned in this review. A manual search of original and review publications was performed to find more relevant studies.

### 2.2. Study Inclusion and Exclusion Criteria

The inclusion criteria for the studies were as follows:

- RA or induced experimental models of inflammatory arthritis (e.g., adjuvant arthritis, collagen-induced arthritis);
- Natural bioactive products, plant-based extracts, phytochemicals, nutraceuticals, or other biologically derived compounds relevant to RA;
- Investigated natural bioactive compounds alone and/or in combination with MTX as appropriate; molecular, biochemical, inflammatory, immunological, oxidative-stress and antioxidant outcomes, and cartilage or bone outcomes relevant to the pathogenesis of RA; provided mechanistic findings on signaling cascades, inflammatory cytokines, oxidative stress markers, antioxidant defences, MMPs, or other relevant molecular targets;
- Relevant original experimental and clinical research or reviews with sufficient mechanistic or translational background.
- Preferred publications from primary research, where specific mechanistic claims were made. The present reviews were used to give a wider context and to find more relevant information.

This includes material not related to RA or experimental inflammatory arthritis, duplicate publications, and studies with a lack of relevance to the mechanistic emphasis of this review.

### 2.3. Literature Screening and Evaluation

Titles and abstracts identified from the literature searches were screened for relevance to the review topic. The appropriate publications were then assessed for their experimental model, bioactive substance examined, molecular targets, and relevance to RA pathogenesis or treatment. Research was concentrated on adjuvant arthritis and other well-known experimental models of arthritis and the interactions between natural bioactive substances and MTX.

The current work was written in the format of a structured narrative review rather than a systematic review, and thus, no formal PRISMA-based study selection procedure or quantitative meta-analysis was performed. Similarly, no explicit approach for risk of bias ratings was used. Instead, the literature selected was relevant to the mechanistic themes of this review (a priori stated), with a preference for primary experimental evidence supporting particular molecular and biochemical pathways.

### 2.4. Data Extraction and Synthesis of Results

The bioactive ingredient or extract, experimental model, treatment regimen, molecular targets, signaling pathways, inflammatory and oxidative-stress biomarkers, antioxidant responses, and effects on cartilage or bone-related outcomes were extracted. We investigated pathways involved in RA pathogenesis, including NF-κB/MAPK, JAK/STAT, PI3K/Akt and Nrf2/HO-1 signaling, cytokines, MMPs, reactive oxygen species, and endogenous antioxidant defences.

The evidence was synthesized in a narrative form to compare molecular mechanisms and probable therapeutic effects of different natural bioactives. *In vitro* and experimental arthritic studies have been separated from clinical evidence where available. Mechanistic data from adjuvant arthritis studies and natural bioactive compounds combined with MTX were considered separately. Finally, due to the narrative approach of this review, conclusions should be interpreted as a structured synthesis of mechanistically important material and not as a systematic review of all available studies.

## 3. Pathophysiology of Rheumatoid Arthritis

RA is one of the most frequent chronic inflammatory diseases. It is mostly related to joints, but it may also be characterized as a syndrome due to its extra-articular manifestations. RA is also influenced by a series of complex interactions related to genetic vulnerability, environmental factors, immune deregulation, and inflammatory signaling. The key pathophysiology of RA is related to synovitis, hyperplasia of the synovial membrane, pannus formation, and subsequent damage of cartilage and/or erosions of bone [5,27].

Table 1 provides the most important pathways involved in RA pathophysiology, together with associated biomarkers measured by our group in AA in selected papers.

### 3.1. Immune Dysregulation and Cytokine Networks

RA is associated with and initiated by dysregulation of both innate and adaptive immune responses. The activated macrophages, dendritic cells, and fibroblast-like synoviocytes produce large amounts of pro-inflammatory cytokines (interleukin-1β (IL-1β), IL-6, and IL-17A), and transforming growth factor-β (TGF-β), which leads to the formation of synovial inflammation and joint destruction next to the synovium. The presence of IL-15, IL-1, IL-6, TGF-β, IL-12, and IL-23 in synovial tissue is linked to T-cell activation. T cells then cause inflammation by releasing IL-17 and interacting with nearby macrophages [7,32,33]. Macrophage activation is also promoted by T helper cell subsets, basically Th1 and Th17 cells, that again amplify inflammation and osteoclastogenesis, encouraging bone resorption and cartilage degradation [34].

B cells are also involved in RA pathogenesis in another mechanism of autoantibody production, including rheumatoid factor (RF) and anti-citrullinated protein antibodies (ACPAs), leading to immune complex formation and activation of complement pathways. B cells are vital for synovitis and are involved, at least in part, through antigen presentation and secretion of cytokines. B-cell differentiation and proliferation are mediated, in turn, by IL-6, IL-10, B-cell-activating factor (BAFF), and proliferation-inducing ligands in synovial tissue [33,35].

### 3.2. Intracellular Signaling Pathways: NF-κB and MAPKs

Numerous studies focus on identifying the molecular triggers that activate NF-κB in RA because NF-κB is essential to the inflammatory process in RA. It is accepted that pro-inflammatory cytokines like TNF and IL-1 are important, and TNF antagonists are a useful treatment for severe RA. Since TNF and IL-1 are both highly effective NF-κB activators, it is reasonable to believe that the majority of their pro-inflammatory effects in RA are mediated by NF-κB activation. Numerous physiological activities depend on NF-κB activation; therefore, controlling the NF-κB signaling pathway and the genes it triggers is essential for controlling inflammatory responses [10]. In rheumatoid joint tissue, cytokines, including tumor necrosis factor (TNF), are highly expressed and play a major role in inflammation and articular degradation. Therefore, the first cytokine to receive complete validation as a treatment target for RA was TNF [36].

On the other hand, the MAPK signaling pathways, p38, ERK, and JNK are known to regulate cytokine synthesis, synoviocyte proliferation, and immune cell activation. There is evidence of MAPK expression in the synovium of patients with RA and osteoarthritis. It was found that the phosphorylation and activation of these enzymes could be observed only in RA synovium, thus pointing towards its implication in RA pathophysiology [37]. The persistent activation of NF-κB and MAPKs leads to a self-prolonging inflammatory cycle that contributes to synovial hyperplasia, pannus formation, and progressive joint destruction.

### 3.3. Oxidative Stress

**Figure 1:**
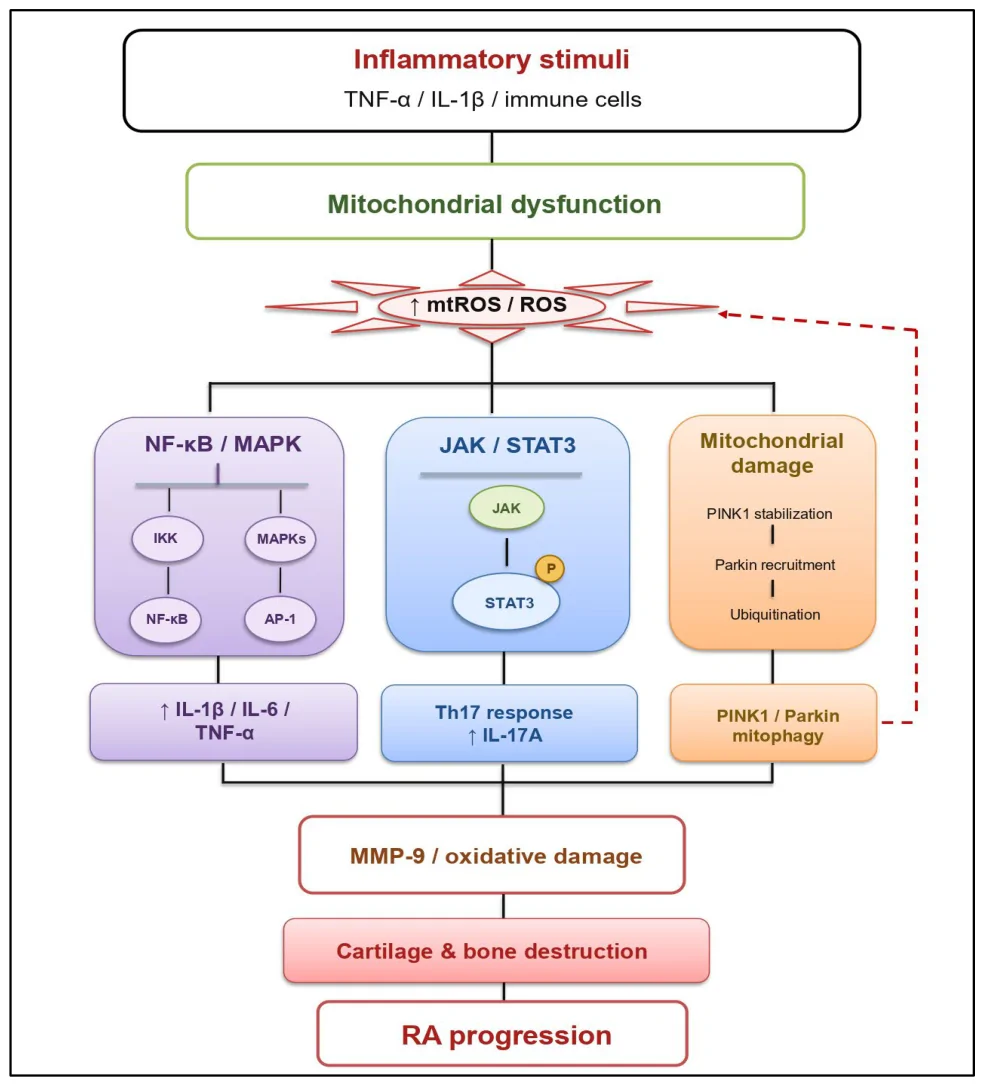
Mitochondrial dysfunction and increased synthesis of mitochondrial and cellular reactive oxygen species (mtROS/ROS) may be caused by inflammatory stimuli such as TNF-α, IL-1β, and activated immune cells. Elevated ROS could increase NF-kB/MAPK and JAK/STAT3 signaling, resulting in enhanced production of pro-inflammatory cytokines and Th17/IL-17A responses. Damaged mitochondria activate PINK1/Parkin-mediated mitophagy as a quality control mechanism. Prolonged oxidative stress and poor mitochondrial homeostasis may further exacerbate oxidative damage and MMP-9-mediated tissue damage, eventually leading to the breakdown of cartilage and bone and progression of RA [46,47,48,49,50,51]. The red dashed arrows represent a vicious cycle between mitochondrial malfunction, mtROS/ROS production and mitochondrial damage, whereas the solid arrows represent the direction of signaling or pathological advancement.

Reactive oxygen species (ROS) are involved in autoimmunity. ROS are significant mediators of cell structural damage during oxidative stress. It is known that the hydroxyl radical reacts with every part of DNA, causing damage to the purine and pyrimidine bases, as well as the deoxyribose backbone. Additionally, oxidation processes can occur in the side chains of amino acids; however, certain amino acids are more sensitive than others. ROS also has an impact on phospholipids, and their polyunsaturated fatty acids are particularly vulnerable to oxidation. Collagens and proteoglycans are examples of matrix molecules that can sustain damage and undergo structural changes when ROS migrate extracellularly [38]. When compared to healthy individuals, whole blood and monocytes produced five times more mitochondrial ROS in RA patients, indicating that OS is a harmful characteristic of the disease. Because they function as secondary messengers in the inflammatory and immunological cellular response in RA, free radicals are indirectly linked to joint destruction. OS in RA is characterized by a discrepancy between ROS/RNS formation. The enzyme superoxide dismutase (SOD) transforms superoxide radicals into hydrogen peroxide (H_2_O_2_), which is detoxified by the enzyme catalase (CAT) and glutathione peroxidases (GPx). GPx is closely correlated with the glutathione system, since glutathione is utilized as a reducing agent in peroxide reduction. Other mechanisms against OS include the expression of antioxidant and cytoprotective proteins regulated by the Nrf2 signaling pathway, such as HO-1 [39,40,41,42]. In clinical and experimental arthritis, the level of glutathione and several antioxidant enzymes, such as glutathione peroxidase and SOD, have been reduced. This could be a result of redox imbalance, which is a major key to maintaining synovitis and accelerating bone and cartilage destruction and/or degradation [43]. Also, excessive ROS formation occurs due to impaired mitochondrial function, which acts as signaling messengers for mitophagy. T-cell exposure to elevated OS can prolong the aberrant immune response and make them resistant to many stimuli, such as those for growth and death. However, free radicals can directly damage joint cartilage by destroying its proteoglycan and preventing its formation [44,45]. Figure 1 shows a schematic overview of the ROS-mediated inflammatory and mitochondrial pathways involved in rheumatoid arthritis progression [46,47,48,49,50,51].

### 3.4. Cartilage Degradation and Bone Erosion

Although RA is an autoimmune disease that affects the joints, it differs from other autoimmune diseases in pathophysiology. Bone is mostly made up of extracellular matrix, which makes it resistant to direct immune system attack. It was first suggested that matrix-degrading enzymes, like MMPs, play a crucial part in the breakdown of bone and cartilage, but the receptor activator of nuclear factor kappa-B ligand (RANKL) expressed by synoviocytes and T cells promotes osteoclast differentiation, leading to bone erosion as well [52]. Osteoblasts release a soluble factor that induces isolated osteoclasts to cause bone resorption when exposed to bone-resorbing substances [53]. Increased osteoclastogenesis is caused by the aberrant expression of RANKL. New therapeutic targets can be immunoreceptor tyrosine-based activation patterns and signaling cascades, including RANKL, in RA [52].

### 3.5. Experimental Model for Studying RA Pathophysiology

To better understand the mechanisms behind constant inflammation in synovial tissue, animal models of arthritis can be useful. They are widely used in the research of new RA therapy approaches. Adjuvant arthritis (AA) can be induced by intradermal or footpad injection of heat-killed mycobacterial species in susceptible rodent strains such as Lewis and Sprague-Dawley rats [44]. Accumulation of mononuclear cells in connective tissues near periosteal surfaces is an early histological feature. Radiographic signs of inflammation may appear approximately 10 days after induction and include periosteal responses, localized osteoporosis, and erosive lesions. Synovial infiltration can promote pannus formation, cartilage deformation, and joint damage [54].

The MAPK/NF-κB inflammatory signaling axis has been investigated in AA induced by complete Freund’s adjuvant in rats. Upregulation of MAPK signaling can enhance NF-κB activation and downstream inflammatory responses [55,56]. In AA, activation of NF-κB and MAPKs is accompanied by redox imbalance, including reduced glutathione levels and increased lipid peroxidation in plasma and tissues. These features make AA useful for studying inflammatory–oxidative crosstalk and for evaluating natural bioactive compounds and MTX combination strategies. However, AA does not fully reproduce the heterogeneity or chronicity of human RA; therefore, findings from AA should be interpreted as mechanistic and preclinical evidence rather than direct clinical correlates [26,57,58,59]. Furthermore, AA can also be used to investigate changes in Janus kinase–signal transducer and activator of transcription (JAK-STAT) signaling associated with inflammatory and oxidative-stress pathways. Oxidative stress may enhance cytokine receptor signaling and sustain STAT activation in this experimental model [60,61].

**Figure 2:**
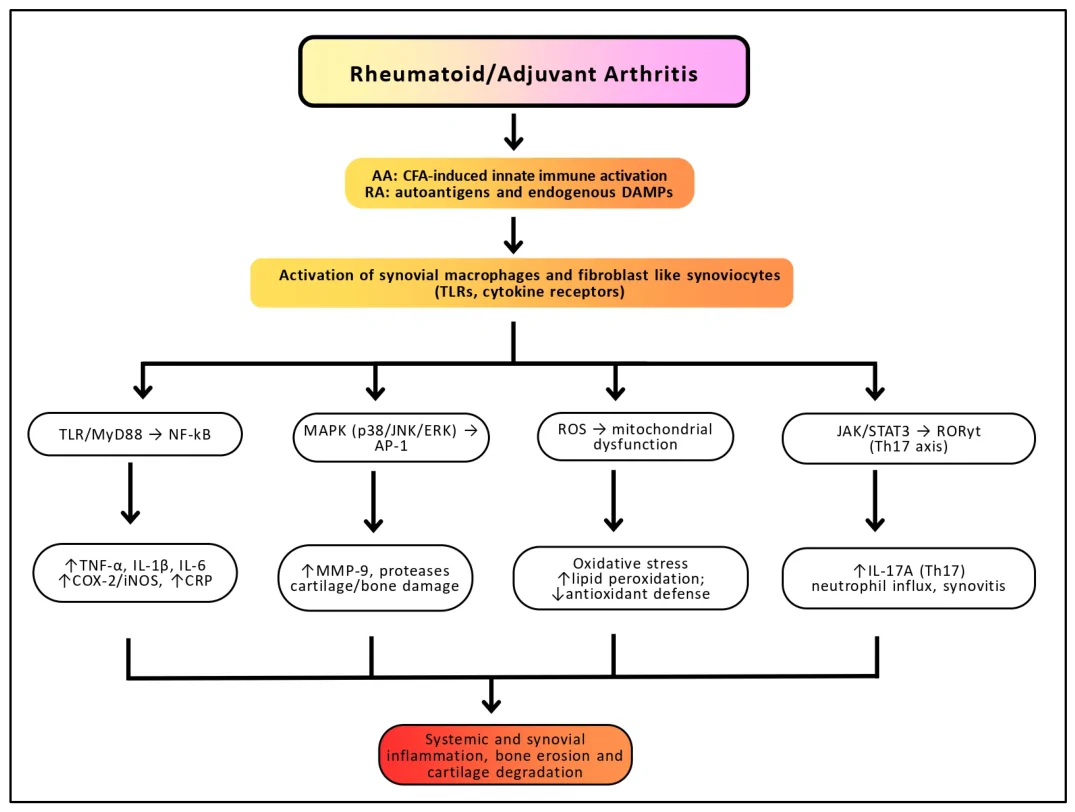
Important pathogenic processes contributing to synovitis, systemic inflammation, cartilage degradation, and bone erosion in rheumatoid arthritis and experimental adjuvant arthritis. The aetiology of RA and AA is initiated by multiple factors, yet several inflammatory and molecular mechanisms are interconnected. In RA, autoantigens and endogenous damage-associated molecular patterns (DAMPs) enhance the activation of synovial macrophages and fibroblast-like synoviocytes (FLSs), whereas complete Freund’s adjuvant (CFA) largely stimulates innate immune activation in AA. These stimuli activate major inflammatory and stress pathways, including TLR/MyD88/NF-κB, MAPK, ROS-related mitochondrial dysfunction, and JAK/STAT3-dependent Th17 signaling. Activation results in release of pro-inflammatory cytokines and mediators, oxidative stress, matrix-degrading enzymes, and IL-17A, leading to persistent synovitis and systemic inflammation, cartilage breakdown, and bone erosion [10,26,27,32,33,34,36,37,44,55,59]. Adjuvant arthritis (AA); complete Freund’s adjuvant (CFA); danger-associated molecular patterns (DAMPs); fibroblast-like synoviocytes (FLSs); mitogen-activated protein kinase (MAPK); matrix metalloproteinase (MMPs); reactive oxygen species (ROS). ↑-increase/elevate; ↓-decrease/suppress.

Figure 2 shows the most important pathogenic processes contributing to synovitis, systemic inflammation, cartilage degradation, and bone erosion in rheumatoid arthritis and experimental adjuvant arthritis. The convergence of these pathways [10,26,27,32,33,34,36,37,44,55,59] ends with continuous synovial inflammation, progressive cartilage degradation, and joint damage.

## 4. Natural Bioactive Compounds in Rheumatoid Arthritis

Natural bioactive compounds that are obtained from plant, food, and/or biological origin are receiving increased attention for their corresponding role in the treatment of RA. Unlike original synthetic drugs, these natural products display pleiotropic effects and cater to the simultaneous control of various pathological processes in RA: inflammation, oxidative/nitrosative stress, immune dysregulation, and tissue destruction. Among them, *Acanthopanax senticosus* HARMS (ASH) and ASH extracts are classified for their pharmacological effects, including neuroprotection and immune modulation [62,63], while *Rhodiola rosea* L. extracts (RSEs) are mostly recognized for their adaptogen characteristics. Eleutheroside E from *Acanthopanax senticosus* reduces the severity of disease by inhibiting NF-κB activities, infiltration of inflammatory cells, pannus formation, cartilage degradation, and bone erosion in collagen-induced arthritis (CIA) mice and decreases the production of TNF-α and IL-6 [64]. Other phenolic constituents of *A. senticosus*, including syringin (eleutheroside B), isofraxidin, and (+)-syringaresinol-di-O-β-D-glucoside, have been shown to exert anti-inflammatory activity by modulating IL-1β-induced responses in synovial cells via AP-1/NF-κB signaling and regulation of inflammatory mediators and MMPs. In addition to these RA-relevant properties, syringin has also been associated with enhanced insulin sensitivity and cardioprotective action, and isofraxidin has been shown to have regulatory effects on lipid metabolism and anticancer activity in animal tests [65,66].

Chlorogenic acid is known as an antioxidant with various proven medical properties [28,67,68,69,70,71,72]. Chlorogenic acid possessed an antiarthritic action in CIA mice, and TNF-α enhanced human synoviocytes by regulating NF-κB signaling to downregulate BAFF expression. In experimental RA, chlorogenic acid has also demonstrated the ability to modulate T-cell responses via AhR/NF-κB signaling and to decrease macrophage inflammatory polarization by modulating the gut microbiota–indolelactic acid—immune axis [73,74]. All these data provide evidence for focused action of selected natural compounds on molecular targets relevant to RA rather than generic anti-inflammatory effects. However, most mechanistic evidence is still preclinical, and translation to human RA is limited by differences between experimental arthritis models and human disease, variability in extract composition and standardization, poor or variable bioavailability, uncertainty about optimal dosing, and insufficient information on pharmacokinetic interactions with conventional DMARDs [75]. Therefore, such medications should be seen as potential supplemental possibilities rather than proven replacements to traditional RA therapy, and their clinical efficacy and safety need to be confirmed in well-designed controlled trials.

### 4.1. Plant-Derived Bioactive Compounds

Plant-derived bioactive compounds are the most studied class of natural agents in RA research. In recent years, natural drugs for treating RA have improved impressively. These natural medicines mainly include flavonoids, polyphenols, alkaloids, glycosides, and terpenes [76,77,78,79,80], all of which possess well-documented anti-inflammatory and antioxidant effects. In experimental arthritis models, these compounds tend to consistently suppress synovial inflammation, reduce paw edema, and improve histopathological features of affected joints [28,67].

At the molecular level, the mechanism of anti-inflammatory action of bioactive compounds from plants is mainly the inhibition of the pro-inflammatory cytokine TNF-α and interleukins IL-1β, IL-6, and IL-17. It is notable that most phytochemicals target the signaling mechanisms mediated by NF-κB or MAPKs; thus, the production of pro-inflammatory mediators is suppressed at the transcriptional level. TNF expression is repressed by apigenin, kaempferol, and resveratrol, which are found in various fruits, vegetables, and grains. Flavonoids have been shown to reduce oxidative stress and inflammation, as well as have beneficial effects on cancer, cardiovascular disease, and chronic inflammatory conditions [25,81]. In AA, the conjugated serotonin compound N-feruloylserotonin (N-f-5HT; N-feruloyl-5-hydroxy-tryptamine) and quercetin reduced nuclear factor kappa-light-chain-enhancer of activated B cell (Nf-kB) activation, as assessed by p65-DNA binding in the liver and lung [82,83]. RSE, which is known for having adaptogen properties, effectively reduces the plasma IL-17A levels in AA [28].

Progressive cartilage degradation and bone erosion are the characteristics of RA pathogenesis. Osteoclasts play a key role in bone degradation, which is associated with autoimmune arthritis. Malfunction of RANKL increases osteoclast formation in arthritis. The pathogenesis of RA is complex, involving a wide variety of cells, including T cells, B cells, and macrophages. Fibroblast-like synoviocytes (FLSs) from the intimal lining layer also have a pivotal role in producing inflammatory cytokines, which promote inflammation, and proteases, which initiate cartilage degradation. Rheumatoid FLSs also exhibit various hostile phenotypes that promote invasion into the extracellular matrix, contributing to increased joint degradation [52,84]. Natural biologically active compounds have distinct protective properties for joint architecture, targeting tissue degradation. Phytoestrogen, which is derived from plants, is known to work on various pathways, reducing inflammation, and it can be effectively used as an alternate source of treatment of RA with fewer side effects. Along with various medications, phytoestrogen can prove to be an effective combinatorial drug with DMARDs in the treatment of RA patients. Certain plant-based ingredients work by inhibiting the gene expression of matrix metalloproteinase, hence preventing cartilage degradation [85]. Some bioactive compounds of plant origin have exhibited chondroprotective advantages by targeting specific MMPs instead of a general action on matrix metalloproteinases. For instance, a natural stilbene, resveratrol, markedly downregulated the expression of MMP-1, MMP-3, and MMP-9 in IL-1β stimulated RA-FLS and also inhibited the expression of RANKL, indicating a combined inhibitory effect on cartilage matrix degradation and osteoclast-associated bone resorption [79,86,87]. In a separate study, resveratrol reduced TNF-α-stimulated MMP-3 production in human RA-FLS through the modulation of the phosphoinositide 3-kinase/protein kinase B (PI3K/Akt) signaling pathway [80,87]. Most recently, the plant-derived stilbene, isorhapontigenin was found to inhibit migration and invasion of RA-FLS by inhibiting farnesyl diphosphate synthase-mediated AKT and ERK1/2 signaling, as well as TNF-α-induced MMP-3 expression, with concomitant reductions in synovial inflammation and joint destruction in collagen-induced arthritic mice [88]. *Crocus sativus* L. (saffron) extracts suppress MMP-9 plasma levels, suggesting inhibition of extracellular matrix destruction in AA [29].

Natural substances may influence both inflammatory mediators and pathways involved in tissue degradation. These effects may be relevant to investigations of mechanisms underlying joint damage and to the development of adjunctive strategies, although their ability to prevent irreversible joint damage in patients with RA remains to be established.

### 4.2. Nutraceuticals and Diet-Derived Compounds

Nutraceuticals and bioactive compounds of dietary origin are other types that are of great interest with respect to having a therapeutic effect in RA. This category of compounds comprises carotenoids, polyunsaturated fatty acids, polysaccharides, and other micronutrient-related compounds that affect inflammatory and immune functions. Their potential application in RA treatment and management could be attributed to their promising safety and systemic bioactivity. Fatty acids have anti-inflammatory properties through diverse mechanisms, many of which are related to or can be attributed to modifications of cellular membrane lipids. These modifications can affect membrane fluidity, signaling for gene expression, and generation of lipid mediators [89,90]. Polyunsaturated omega-3 fatty acids (ω-3-PUFA) and coenzyme Q_10_ reduced inflammation and increased antioxidant activity in AA [91].

In experimental arthritis models, selected nutraceuticals have been reported to reduce pro-inflammatory cytokines, modulate immune-cell activation, and support endogenous antioxidant defenses. These effects may involve suppression of TNF-α, IL-1β, and IL-6 and modulation of macrophage and T-cell responses in inflamed joint tissues [7,92]. Among the compounds with established potent antioxidant properties in the scavenging of ROS are carotenoids and polyphenolic compounds. Dietary phenolic substances such as flavonoids, carotenoids and alkaloids have been shown to reduce the progression of arthritic illness by influencing prooxidant and pro-inflammatory pathways [92,93]. Astaxanthin, sesame oil, carnosine, green tea extract (polyphenols), and hyaluronan mediate the enhancement of the body’s antioxidant defense mechanisms through the upregulation of antioxidant enzymes. They inhibit inflammatory processes and protect the cartilage from damage in the event of an arthritic attack in AA [8,12,30,94,95,96,97,98].

### 4.3. Bee-Derived and Other Biologically Sourced Compounds

Bee venom and apitherapy have been investigated as complementary approaches for inflammatory diseases, including RA, but should not be referred to as established treatments. Apitherapy is the use of bee products such as pollen, propolis, honey, royal jelly, and bee venom. Bee products contain bioactive substances with immunomodulatory properties. Melittin is one of the major components of bee venom and has been investigated for its anti-inflammatory, antimicrobial, and antitumour activities [99,100,101,102,103,104]. Experimental studies have reported the effects of melittin and bee-venom preparations on inflammatory responses in several tissues. In a CIA rat model, a water-soluble component of bee venom reduced paw edema, radiographic changes, IL-6 levels, and nociceptive responses [101,105]. Also, endogenous unconjugated bilirubin has also been reported to exert effective antioxidant and immunomodulatory effects in AA [106]. These findings remain predominantly preclinical and do not establish melittin or bee venom as clinical RA therapies.

Bee venom and its bioactive compounds regulate not only innate immunity but also adaptive immunity via the regulation of macrophage inflammatory signaling and T-cell-mediated immune responses, which contribute to maintaining the homeostasis of the immune system [107,108]. Apart from biological potency, they also have adverse effects. For this reason, the bee venom dose needs to be carefully optimized to reduce these side effects and improve therapeutic effects [109].

### 4.4. The Adjunctive Use of Natural Substances in Rheumatoid Arthritis Therapy

The multifunctional properties of natural bioactive extracts and compounds provide a biological rationale for their potential use alongside established antirheumatic therapies. These compounds may interfere with inflammation signaling, oxidative stress, immunological responses and tissue-destructive processes, while their potential cardioprotective and neuroprotective effects may also be relevant to the extra-articular manifestations of RA [110,111,112,113].

The anti-inflammatory and antioxidant effects summarized in Table 2 have been mainly studied in experimental models of arthritis, AIA, and CIA, which are useful mechanistic platforms for evaluating natural compounds as monotherapies or in combination with DMARDs such as MTX prior to clinical translation. However, preclinical evidence is not uniformly encouraging and seems to differ according to the outcome, dose, formulation, and treatment conditions. For example, pure *Rhodiola rosea* extracts exhibited modest efficacy on various inflammatory indicators in AA, while the combination of RSE with MTX resulted in improved chosen outcomes but did not prove to be consistently superior to MTX alone on all parameters [28]. Similarly, the effectiveness of curcumin in adjuvant-induced arthritis varied according to its formulation, showing that efficacy cannot be anticipated from nominal dose alone [112,114,115].

The clinical evidence is also diverse. A recent systematic review and meta-analysis did not find significant overall effects of curcumin or *Curcuma longa* extracts on several RA-related outcomes. However, another meta-analysis of placebo-controlled RA trials reported improvements in selected clinical and inflammatory parameters, although with low or very low certainty of evidence. These discrepancies could be related to differences in formulation, dose, treatment duration, patient characteristics, and study design. Moreover, bioavailability, dose optimization and standardization are still limiting factors for the translation of encouraging experimental results into evidence-based RA therapy [128,131,132]. Importantly, clinical evidence is available for natural bioactive compounds as adjuncts to MTX, though it remains considerably less extensive and more heterogeneous than the preclinical literature. A potential benefit of omega-3 fatty acids has also been demonstrated in patients receiving MTX-based therapy. In a randomized double-blind trial of recent-onset RA, high-dose eicosapentaenoic acid, and docosahexaenoic acid (5.5 g/day) with MTX, sulfasalazine and hydroxychloroquine reduced treatment failure and increased remission rates compared with low-dose supplementation [133]. The clinical data indicating vitamin D supplementation is more inconsistent. In patients with active RA receiving stable doses of MTX, vitamin D supplementation (50,000 IU/week for 12 weeks) did not significantly improve disease activity score responses compared to placebos. A randomized study where a single dose of 300,000 IU of cholecalciferol (vitamin D3) was added to MTX and glucocorticoid therapy reported improvements in selected patient and immunological outcomes but could not demonstrate a significant additional improvement in disease activity scores. Thus, the clinical response to vitamin D supplementation might depend on baseline vitamin D status, dose, treatment duration, and the outcome assessed [134,135]. Evidence is available for compounds such as omega-3 fatty acids and vitamin D; however, the available studies are limited and heterogeneous, and their findings are not always directly applicable to the evaluation of MTX-based combination strategies.

*Tripterygium wilfordii* Hook F has relatively stronger clinical evidence among plant-derived agents. In a randomized trial, the combination of *T. wilfordii* and MTX resulted in a greater response compared to MTX alone, and a meta-analysis of six randomized trials that included 643 patients also showed enhanced clinical responses with the combination [136,137]. The current findings should be considered encouraging but not conclusive, highlighting the necessity of standardized preparations, well-conducted studies, clinically relevant outcomes, and pharmacokinetic evaluation to determine replicable effects of natural substances as adjuncts to MTX. However, challenges related to bioavailability, dose optimization, and standardization are key considerations for the successful integration of natural substances into evidence-based RA therapy.

## 5. Molecular Mechanisms of Natural Bioactive Compound Actions in Rheumatoid Arthritis

Natural bioactive compounds can influence the pathways of inflammation, oxidative stress, immune response, and tissue destruction rather than targeting a specific molecular unit in RA. Many of these molecular mechanisms have been studied in animal models of arthritis such as adjuvant arthritis, which strongly shows synovial inflammation, cytokine surge, oxidative stress, and activation of NF-κB/MAPK signaling. These properties make it easier for mechanistic validation and for choosing a combination therapy (e.g., with methotrexate) [26,138,139]. However, mechanistic findings from AA should be distinguished from evidence in human RA.

### 5.1. Modulation of Pro-Inflammatory Cytokine Networks

One of the key mechanisms through which natural compounds reduce RA pathology is suppression of pro-inflammatory cytokine production and an increase in anti-inflammatory cytokines. Increased levels of interleukins IL-1β, IL-6, IL-17, and TNF-α play a pivotal role in supporting synovial inflammation, cartilage and bone degradation, and immune-cell recruitment. In preclinical arthritis models, such as adjuvant arthritis, natural compounds have been shown to reduce the expression/overexpression and release of these cytokines [7,140]. Biological DMARDs induce TNF inhibition, interleukin-6 receptor inhibition, T-cell costimulation blockage, and B-cell depletion. These four mechanisms of action, among others, were described as approved biological treatments for RA by Smolen et al. [5]. However, only a limited percentage of patients react to IL-1 pathway blockage. Five TNF inhibitors are currently approved: four for subcutaneous administration (adalimumab, certolizumab pegol, etanercept, and golimumab) and one for intravenous use (infliximab) [5].

Since p38 mitogen-activated protein kinase (p38 MAPK), an important signal transduction pathway of inflammation, is thought to be essential for the induction and maintenance of chronic inflammation, its elements become potential molecular targets of small-molecule inhibitors for the control of inflammation. On the other hand, the NF-κB family of transcription factors is necessary for the development of pro-inflammatory cytokines, but it can also trigger regulatory pathways. There are two different ways to activate NF-κB: the classical or canonical pathway and the alternative or non-canonical pathway. Both acute inflammatory responses and chronic inflammatory disorders, such as RA, are known to depend on the canonical NF-κB pathway. Natural bioactives block NF-κB nuclear translocation and the transcription of inflammatory genes by interfering with the phosphorylation and degradation of inhibitor κB (IκB). Additionally, ERK and JNK contribute to decreased inflammatory signaling and cytokine production [139,141].

### 5.2. Regulation of Cyclooxygenase and Nitric Oxide Pathways

Cyclooxygenase-2 (COX-2) and inducible nitric oxide synthase (iNOS) are key enzymes involved in inflammatory mediators that cause inflammation in RA. COX-2 overexpression leads to the formation of excessive amounts of prostaglandin E2 synthesis, which contributes to the formation of synovial hyperplasia, pain, and edema. Nevertheless, enhanced iNOS activity determines the elevated level of nitric oxide and increases nitrosative stress within inflamed joints [142,143].

Natural bioactive compounds have revealed the capability of suppressing COX-2 and iNOS expression at both transcriptional and protein levels, primarily through the upstream inhibition of NF-κB and MAPK signaling. This dual nature of suppression reduces prostaglandin and nitric oxide production, hence decreasing inflammatory responses and tissue injury. Such combined inhibitions show that many natural compounds can contribute to a strong therapeutic outcome [142,143,144,145].

### 5.3. JAK-STAT Signaling

The Janus kinase-signal transducer and activator of transcription (JAK-STAT) pathway is a key mediator of cytokine-driven inflammation in RA. Pro-inflammatory cytokines, including IL-6, IL-12, and IL-23, as well as interferons and granulocyte-macrophage colony-stimulating factor, signal mainly through this pathway, promoting immune activation, synovial inflammation, and joint destruction. The clinical success of targeted synthetic disease-modifying antirheumatic drugs (tsDMARDs) has safely shown JAK-STAT signaling as a proven therapeutic target in RA. tsDMARDs include tofacitinib, baricitinib, and upadacitinib [146,147,148].

In addition to direct pharmacological inhibition, promising evidence suggests that natural bioactive compounds may indirectly lower JAK-STAT signaling through upstream mechanisms rather than working as a direct JAK inhibitor. These compounds first suppress cytokine formation, mitigate the receptor-proximal signaling, and also regulate redox-sensitive pathways, which help in STAT activation. In STAT family members, STAT3 is especially significant in RA pathogenesis because of its function in synovial fibroblast activation, Th17 cell differentiation, angiogenesis, and continuous inflammatory gene expression [149,150,151].

Alterations in JAK-STAT signaling have also been studied in experimental models of RA, such as AA. AA reproduces several key cytokine-dependent and oxidative-stress-driven mechanisms observed in human RA, such as enhanced IL-6 signaling, sustained STAT3 phosphorylation, and increased transcription of inflammatory mediators in synovial tissues [25,26,30,152]. Nevertheless, these similarities should be interpreted as mechanistic similarities at the level of the model, not as proof that AA is a perfect replication of human RA.

### 5.4. Integrated Multitarget Effects and Therapeutic Implications of Natural Compounds

Bone erosion and cartilage degradation are characteristics of RA, and they contribute to irreversible joint damage. Natural bioactive compounds exert shielding effects on joint structure by diminishing matrix metalloproteinases (MMPs). MMPs are enzymes that are responsible for extracellular-matrix degradation and pannus invasion. Suppression of MMP demonstrates cartilage integrity and controls synovial tissue invasiveness [84]. In addition, several natural compounds interfere with receptor activator of nuclear factor-κB ligand (RANKL) signaling, thus inhibiting osteoclast proliferation and activity. These compounds slow down or decrease bone resorption and erosion through interfering with osteoimmunological pathways, contributing to long-term joint protection [52].

Natural bioactive compounds with antioxidant and anti-inflammatory properties can weaken STAT-dependent transcription by reducing cytokine availability, preventing NF-κB activation, and also reestablishing redox homeostasis through the nuclear factor erythroid 2-related factor 2 (Nrf2) pathway. By reducing oxidative stress, these compounds indirectly inhibit cytokine-driven JAK-STAT signaling and downstream expression of genes that are involved in inflammation, angiogenesis, and tissue destruction [25,152].

From a therapeutic point of view, modulation of JAK-STAT signaling by natural bioactive compounds should be considered as complementary to pharmacological JAK inhibition achieved by synthetic drugs. JAK inhibitors have shown suppression of inflammatory signaling, but their long-term use is associated with safety concerns. In contrast, natural compounds apply broader but milder regulatory effects across inflammatory and oxidative pathways. Their indirect interaction with JAK-STAT signaling may be very relevant in multitarget and combination therapies targeted at reducing inflammatory problems while minimizing drug-associated toxicity [25].

All these mechanisms indicate that natural bioactive compounds can influence inflammatory signaling, oxidative stress, immune dysregulation, and tissue-destructive processes simultaneously in RA. This combined, multitargeted approach of action provides a strong mechanistic foundation for their employment as adjunct therapies in conjunction with traditional antirheumatic medications, especially methotrexate. Mechanistically, the AA model provides a proven preclinical system to investigate the above-stated pathway-level actions, since it faithfully mimics several important inflammatory, oxidative, and immunologic aspects of RA. Thus, the results of AA must be considered mechanistic/preclinical and suggestive of translational potential and should not be equated with clinical efficacy in RA [8,30,44].

## 6. Combination Therapy with Methotrexate: Preclinical Evidence for Complementary Effects of Natural Bioactive Compounds

Methotrexate (MTX) remains a first-line disease-modifying antirheumatic drug for RA, and it is well prescribed not only as a monotherapy but also as a combination therapy with other antirheumatic agents or natural compounds. The primary mechanism for MTX is its involvement in the inhibition of folate-dependent enzymes, suppression of purine and pyrimidine synthesis, and inflammatory signaling pathway modulation, eventually leading to reduced immune-cell proliferation and cytokine production [15]. Despite having proven efficacy, MTX therapy is commonly limited by adverse effects, including hepatotoxicity, gastrointestinal intolerance, hematological disturbances, and oxidative-stress-related tissue injury, and a significant proportion of patients exhibit incomplete or short clinical responses [153,154].

These limitations have contributed to an increasing interest in the combination of methotrexate with natural bioactive compounds, like curcumin, resveratrol, quercetin, epigallocatechin gallate (EGCG), β-cryptoxanthin, and bee-derived peptides such as melittin, as a potential strategy to improve treatment responses and reduce treatment-associated toxicity. Pharmacokinetic limitations restrict the clinical use of natural bioactive substances, such as curcumin and resveratrol. Although they are recognized for their anti-inflammatory and antioxidant activities, their oral bioavailability is limited by their poor solubility, extensive metabolism, and unpredictable systemic exposure. Regarding curcumin, differences in absorption and biological activity depending on formulation may also explain variations reported in experimental trials, as indicated by the different effects of curcumin formulations in experimental arthritis. Moreover, the extensive metabolism and poor oral bioavailability of resveratrol can limit the translation of preclinical findings into clinical practice. Pharmacokinetic challenges may play a role in the variations between preclinical and clinical outcomes [131,132,155]. Additionally, variables including formulation, dosage, duration of treatment, patient characteristics, severity of illness, and study design may also influence therapeutic benefits. Experimental evidence from arthritis models, particularly AA, shows that these compounds may potentiate the anti-inflammatory effects of MTX through complementary mechanisms [17,110,112].

### 6.1. Enhancement of Anti-Inflammatory Efficacy

Natural bioactive compounds such as curcumin, resveratrol, and quercetin can increase the anti-inflammatory effects of MTX by suppressing pro-inflammatory cytokines including TNF-α, IL-1β, IL-6, and IL-17. MTX’s mechanism of action lies largely in its ability to impair the proliferation and cytokine production by immune cells, whereas these drugs exert their effect by modulating the signaling cascades, particularly the NF-κB and MAPK pathways, with a more apparent impact on reducing inflammation within the joint microenvironment. The combination therapies have been found to affect synovial hypertrophy and inflammation more specifically than MTX in experimental animals with arthritis [30,75,83,84,87,110,113,139,156,157]. The probiotic *Lactiplantibacillus plantarum* LS/07 and ferulaldehyde improved the effectiveness of MTX by increasing the antioxidant status and inhibiting inflammatory mediators such as IL-17A and MMP-9 in AA [158,159]. In AA, selenium-enriched *Enterococcus faecium* potentiated the effects of MTX by suppressing paw swelling, arthritic and radiographic scores, and bone mineral loss. The probiotic *Escherichia coli* O83 exerted a complementary effect on inflammatory and arthritic changes in AA [160,161]. These findings support further preclinical investigation but do not establish improved clinical efficacy in patients with RA.

### 6.2. Reduction in Methotrexate-Induced Toxicity

Oxidative stress is a key factor in adverse effects of MTX, specifically hepatotoxicity and gastrointestinal injury [16]. Antioxidant-rich phytochemicals such as resveratrol, quercetin, carotenoids (β-cryptoxanthin and astaxanthin), and polyphenols can counter oxidative and nitrosative stress induced in MTX toxicity through endogenous antioxidant pathways, such as SOD, CAT, glutathione peroxidase, and Nrf2/HO-1. By this, redox homeostasis is normalized, and non-target tissues will be protected from oxidative damage, thus possibly reducing MTX toxicity and supporting sustained therapy [12,94,96,122,123,157,162]. Hyaluronic acid and carnosic acid, astaxanthin, β-(1,3/1,6)-d-glucan isolated from *Pleurotus ostreatus* (β-glucan-PO), and robinin have shown potential to reduce the adverse effects of MTX in AA [30,96,97,163].

### 6.3. Multitarget Modulation of Immune and Redox Pathways

The enhanced effects observed in MTX with natural compound combinations could be attributed to their complementary and multi-target actions. MTX mainly affects cell proliferation of immune cells and production of inflammatory mediators, whereas naturally active agents affect four different pathways: correction of oxidative stress, activation of immune cells (macrophages and T cells), mitochondria, and tissue-destructive enzymes. This multitarget change addresses various pathological factors of RA simultaneously and helps improve disease control at possibly lower MTX doses [5,30,96,97,119,158,164]. Table 3 summarizes studies of MTX combinations with selected natural extracts and compounds, along with biological outcomes and observed pathway changes in experimental arthritis.

### 6.4. Translational and Clinical Implications

The combination of natural bioactive compounds with methotrexate-based therapeutic treatments shows a promising approach for improving RA management. However, although preclinical evidence supports translation into clinical practice, careful understanding of dose optimization, pharmacokinetic interactions, standardization of natural compounds, and long-term safety is needed. Pharmacokinetic interactions represent an additional consideration when natural bioactive compounds are combined with MTX. Natural compounds can affect drug exposure by modulating drug-metabolizing enzymes and membrane transporters involved in absorption, distribution, and elimination. Curcumin has been shown to interact with several drug disposition pathways, including P-glycoprotein (P-gp), breast cancer resistance protein, and other membrane transporters. However, the direction and magnitude of these effects depend on dose, formulation, exposure, and experimental context, and their clinical relevance is unclear [165]. Also, resveratrol has additionally demonstrated a potential for interaction with drug disposition pathways, although the current evidence is mostly experimental and the magnitude and clinical relevance of such interactions are not yet well established [166].

An especially relevant example in the case of combinations of natural compounds and MTX is the extract of *Rhodiola rosea.* The RSE in combination with a subtherapeutic dose of MTX in experimental AA exerted additional effects on selected inflammatory parameters, including reductions in plasma CRP and MMP-9. However, the combination did not consistently produce better results than MTX alone across clinical endpoints [28]. RSE demonstrated potent *in vitro* inhibition of cytochrome P450 3A4 (CYP3A4)-mediated metabolism and P-glycoprotein (P-gp) efflux transport (IC_50_ = 1.7–3.1 μg/mL for CYP3A4 and 16.7–51.7 μg/mL for P-gp) [167]. This distinction is important because MTX disposition is largely determined by membrane transporters involved in cellular uptake and efflux and not solely by CYP-mediated metabolism. The transporter families have been shown to play a role in MTX disposition, efficacy, and resistance in RA [168].

These observations support the consideration of potential pharmacokinetic interactions but should not be interpreted as evidence that curcumin or resveratrol necessarily alter MTX exposure in patients with RA. Well-designed clinical trials are essential to confirm the effectiveness and safety of specific MTX natural compound combinations before evidence-based suggestions can be established [115,169].

## 7. Conclusions

The evidence discussed in this review article demonstrates that natural bioactive extracts/compounds can modulate several interlinked pathological processes in RA, including inflammatory cytokine signaling, oxidative and nitrosative stress, immune-cell activation, matrix remodeling, and tissue destruction. The adjuvant arthritis model has been particularly beneficial for mechanistic validation because it reproduces a number of inflammatory, oxidative, and tissue-destructive processes relevant to RA and enables simultaneous assessments of systemic biomarkers and joint pathology. Studies of AA have shown that referred compounds can modulate the Th17/IL-17 axis, NF-κB/MAPK signaling, redox-sensitive Nrf2 pathways, and matrix-remodeling markers such as MMP-9. These findings should be considered as evidence from an experimental model and not as direct confirmation of clinical relevance.

Among the bioactive compounds investigated in preclinical models, several have shown potential to complement methotrexate (MTX) therapy. Studies using adjuvant arthritis (AA) models have reported beneficial effects of astaxanthin, *Rhodiola rosea extract*, saffron-derived preparations, carnosic acid, CoQ10, hyaluronan, and robinin, particularly in reducing inflammatory responses and oxidative stress. However, these findings are largely mechanistic and derived from experimental models; therefore, they should not be interpreted as evidence of clinical efficacy in rheumatoid arthritis.

Despite promising preclinical findings, several issues continue to constrain their clinical translation. Evidence for most natural compounds remains profoundly dependent on animal studies, with relatively limited validation in rigorous clinical trials using standardized and chemically characterized preparations. Variability in composition, extraction procedures, dosing, bioavailability, and pharmacokinetic behavior further complicates interpretation across studies. Moreover, the extent to which the gut–joint axis and microbiota-derived metabolites contribute to the observed effects remains unclear, and many proposed molecular and microbial mechanisms still require causal validation. Addressing these limitations will require integrated studies combining pharmacokinetic and pharmacodynamic characterization with biomarker-guided approaches, microbiome–metabolite profiling, and adequately powered randomized trials, alongside careful evaluation of long-term safety and interactions with MTX. Validation in complementary arthritis models will also be important, as adjuvant arthritis does not capture the full immunological heterogeneity of human RA, ultimately helping to identify which experimental effects are robust enough to warrant clinical translation.

## Figures and Tables

**Table 1:** Pathway, associated biomarkers in AA, and treatment-induced modulation.

Pathway Node	Disease Associated Biomarkers in AA	Experimental Model and Treatment Regimen	Treatment-Associated Modulation	References
**Th17 axis**	↑ IL-17A	**AA, Lewis rats:** RSE 150 mg/kg/day + MTX 0.3 mg/kg twice weekly, 21 days; SF 25 or 50 mg/kg/day ± MTX 0.3 mg/kg twice weekly, 21 days; SHA 0.5 mg/kg/day + MTX 0.3 mg/kg twice weekly, 28 days	↓ IL-17A with RSE + MTX; ↓ IL-17A with SF alone and SF + MTX; ↓ IL-17A with SHA + MTX (plasma)	[28,29,30]
**NF-κB/pro-inflammatory cytokines**	↑ IL-1β, ↑ IL-6, ↑ CRP	**AA, Lewis rats:** SF 25 or 50 mg/kg/day ± MTX 0.3 mg/kg twice weekly, 21 days; RSE 150 mg/kg/day + MTX 0.3 mg/kg twice weekly, 21 days	↓ IL-1β (plasma and liver mRNA) with SF; ↓ IL-6/CRP (plasma) with RSE + MTX	[28,29]
**Tissue remodeling/MMPs**	↑ MMP-9	**AA, Lewis rats:** RSE 150 mg/kg/day + MTX 0.3 mg/kg twice weekly, 21 days; SF 25 or 50 mg/kg/day ± MTX 0.3 mg/kg twice weekly, 21 days	↓ MMP-9 with RSE + MTX; ↓ MMP-9 with SF alone and SF + MTX	[28,29]
**Oxidative stress (ROS ↔ Nrf2 defenses)**	↑ oxidative stress; ↓ antioxidant capacity	**AA, Lewis rats:** CoQ10 20 mg/kg/day + MTX 0.3 mg/kg twice weekly; SHA/VHA 0.5 mg/kg/day + MTX 0.3 mg/kg twice weekly, 28 days	CoQ10 + MTX improved oxidative stress parameters; HA reduced ROS	[30,31]

RSE—*Rhodiola rosea* L. extract; MTX—methotrexate; SF—saffron; SHA—Stredná hyaluronan (medium molecular weight); ↑- increase/elevate; ↓ decrease/suppress.

**Table 2:** Natural bioactive extracts/compounds tested in experimental arthritis.

Natural Bioactive Class/Source	Representative Compounds/Products	Experimental Model	Dose/Treatment Regimen	Treatment Duration	Major Molecular Targets in Plasma and Tissues	Major Therapeutic Outcomes	References
**Plant-derived polyphenols/flavonoids**	Genkwanin; naringin; β-sitosterol-containing preparation; robinin	CFA/AIA rat models	Genkwanin: 10 mg/kg/day; Naringin: 25 mg/kg/day; β-sitosterol: 2.5 or 25 mg/kg; Robinin: 50 mg/kg/day	Genkwanin: NR; Naringin: 14 days; β-sitosterol: 28 days; Robinin: 28 days	↓ TNF-α, IL-1β, IL-6 and IL-17; modulation of JAK/STAT, NF-κB, MAPK and Nrf2/HO-1 pathways	Reduced synovial inflammation and oxidative stress; attenuation of arthritis-associated tissue damage	[116,117,118,119]
**Phenolic** **compounds**	Perillyl alcohol; dehydrozingerone	CFA/AIA rat models	Perillyl alcohol: 100 or 200 mg/kg; Dehydrozingerone: 100 mg/kg	Perillyl alcohol: 28 days; Dehydrozingerone: 28 days	↓ COX-2, iNOS, NO and PGE_2_; modulation of NF-κB/Nrf2-related signaling; ↓ inflammatory and oxidative-stress markers	Decreased paw edema/hyperalgesia, inflammatory responses and joint/tissue injury	[97,120,121]
**Carotenoids**	Astaxanthin; β-cryptoxanthin	CFA/AIA rat models	Astaxanthin: 20 mg/kg/day; β-cryptoxanthin: 0.1–1 mg/kg	Astaxanthin: 28 days; β-cryptoxanthin: 4 days in the AIA cartilage study	↑ SOD, CAT and GPx; ↓ ROS/RNS; modulation of inflammatory mediators and cartilage-degrading processes	Restoration of redox balance; reduced inflammation and protection against cartilage degradation	[96,122,123]
**Bee-derived products**	Melittin; bee venom (BV); bee venom + hesperidin	CFA/AIA rat models	Melittin: 0.1 mg/kg; bee venom: 60 mg/kg/day in systemic BV study; BV: 1 mg/kg/day + hesperidin 25 mg/kg/day	Melittin: 15 days; systemic BV: 21 days; BV + hesperidin: 21 days	Immunomodulation; ↓ macrophage/Th17-associated inflammatory activity; ↓ oxidative stress	Reduced inflammation and improved immune/redox balance	[124,125,126,127]
**Biologically derived/endogenous compounds**	Hyaluronan (HA); carnosic acid	AIA rat models	HA: 0.5 mg/kg/day; Carnosic acid: 100 mg/kg/day	HA: 28 days; Carnosic acid: 28 days	↓ inflammatory cytokines; modulation of IL-17A/MMP-9; antioxidant/redox effects; reduction in MTX-associated oxidative stress	Ameliorated arthritis severity; enhanced anti-inflammatory effects and/or protection against treatment-associated oxidative injury	[30,96,97]
**Curcumin-based formulations**	Different curcumin preparations; curcumin-based hydrogel beads	CFA/AIA rat models	Curcumin: 200 mg/kg, three times/week; MTX in combination study: 0.75 mg/kg twice weekly	4 weeks	↓ inflammatory signaling, oxidative stress and MMPs	Joint preservation and systemic anti-inflammatory effects	[128,129]
**Carnosine and formulations**	Carnosine; liposomal carnosine	AIA rat model	Carnosine: 150 mg/kg/day	28 days	↓ inflammatory cytokines, oxidative stress and IL-1α; ↑ antioxidant defenses	Suppress arthritis severity, inflammation and oxidative stress; improved immune/redox balance	[59,130]

**NR**—not reported; ↑-increase/elevate; ↓-decrease/suppress.

**Table 3:** Extracts/compounds combination with MTX tested in adjuvant arthritis.

Compound/Extract	Comparator	Treatment Regimen	Key Readouts in Biometry, Plasma and Tissues	Observed Pathway Changes	References
Dry standardized *Rhodiola rosea* L. extract (RSE)	RSE alone vs. RSE + low-dose MTX	RSE: 150 mg/kg/day + MTX: 0.3 mg/kg twice weekly; 21 days	↓ hind-paw volume (early); ↓ MMP-9; ↓ IL-17A; ↓ IL-6; ↓ CRP (notably in RSE + MTX)	Inhibits Th17 axis (IL-17A) and downstream inflammatory matrix remodeling (MMP-9)	[28]
*Crocus sativus* L. extract (Saffron; SF)	SF (2 different doses) alone and SF + MTX	SF: 25 or 50 mg/kg/day + MTX: 0.3 mg/kg twice weekly; 21 days	↓ IL-17A; ↓ IL-1β; ↓ MMP-9 (day 21); ↓ CRP (higher SF dose + MTX); ↓ IL-1β mRNA in liver	Inhibits IL-1β/NF-κB inflammatory signaling and Th17-associated cytokine outputs	[29]
Coenzyme Q10 (CoQ10)	MTX vs. MTX + CoQ10	CoQ10: 20 mg/kg/day + MTX: 0.3 mg/kg twice weekly; 28 days	Combination suppressed arthritic progression more than MTX; improved oxidative stress/inflammation parameters measured in plasma and in spleen + joints	Supports mitochondrial redox balance; antioxidant pathway support (ROS ↓/endogenous antioxidants ↑)	[31]
Robinin (*Astragalus falcatus*)	Robinin mono; MTX alone vs. Robinin + MTX	Robinin: 50 mg/kg/day + MTX: 0.3 mg/kg twice weekly; 28 days	MTX + robinin superior: ↓ hind-paw volume; ↓ GGT activity; ↓ IL-17A; improved antioxidant mechanism	Enhances antioxidant and suppresses Th17-associated inflammation, potentiating MTX efficacy	[119]
Astaxanthin	Astaxanthin mono; MTX alone vs. Astaxanthin + MTX	Astaxanthin: 20 mg/kg/day + MTX: 0.3 mg/kg twice weekly; 28 days	MTX + astaxanthin superior: ↓ IL-17A; ↓ MMP-9; ↓ oxidative stress; improved arthritis progression	Potent carotenoid antioxidant; enhances antioxidant defenses and markedly suppresses inflammatory mediators	[96]
*Lactiplantibacillus plantarum* LS/07	LS/07 alone; MTX alone vs. LS/07 + MTX	LS/07: 0.4 × 10^9^ CFU/day + MTX: 0.3 mg/kg twice weekly; 28 days	MTX + LS/07 superior: ↓ hind-paw volume; ↓ IL-17A; ↓ MMP-9; ↓ MCP-1; ↓ GGT activity in hind paw tissue	Modulates gut immune responses, suppresses Th17-associated inflammation and enhances antioxidant mechanisms	[158]
Oral hyaluronan (HA), SHA (0.99 MDa) vs. VHA (1.73 MDa)	HA mono; MTX alone vs. MTX + SHA vs. MTX + VHA	HA: 0.5 mg/kg/day + MTX: 0.3 mg/kg twice weekly; 28 days	MTX + SHA superior: ↓ hind-paw volume; ↓ plasmatic IL-17A; ↓ GGT (spleen & joints)	Modulates Th17 output (↓ IL-17A); HA described to scavenge ROS and reduce MMP/inflammatory mediators	[30]

↑-increase/elevate; ↓-decrease/suppress.

## Data Availability

No new data were created or analyzed in this study. Data sharing is not applicable to this article.

## Abbreviations

The following abbreviations are used in this manuscript:

AA	Adjuvant arthritis
AIA	Adjuvant-induced arthritis
ASH	*Acanthopanax senticosus* Harms
BAFF	B-cell activating factor
BV	Bee venom
CAT	Catalase
CFA	Complete Freund’s adjuvant
CIA	Collagen-induced arthritis
CoQ10	Coenzyme Q10
COX-2	Cyclooxygenase-2
CRP	C-reactive protein
CVD	Cardiovascular disease
CYP3A4	cytochrome P450 3A4
DAMPs	Damage-associated molecular patterns
DMARDs	Disease-modifying antirheumatic drugs
DNA	Deoxyribonucleic acid
EGCG	Epigallocatechin-3-gallate
ERK	Extracellular signal-regulated kinase
FLS	Fibroblast-like synoviocytes
GGT	Gamma-glutamyl transferase
GPx	Glutathione peroxidase
HA	Hyaluronan
HO-1	Heme oxygenase-1
IκB	Inhibitor of nuclear factor kappa B
IL	Interleukin
IL-1β	Interleukin-1 beta
IL-6	Interleukin-6
IL-10	Interleukin-10
IL-12	Interleukin-12
IL-15	Interleukin-15
IL-17	Interleukin-17
IL-17A	Interleukin-17A
IL-23	Interleukin-23
iNOS	Inducible nitric oxide synthase
JAK	Janus kinase
JAK–STAT	Janus kinase-signal transducer and activator of transcription
JNK	c-Jun N-terminal kinase
MAPK	Mitogen-activated protein kinase
MCP-1	Monocyte chemoattractant protein-1
MDA	Malondialdehyde
MMPs	Matrix metalloproteinases
MMP-9	Matrix metalloproteinase-9
MTX	Methotrexate
MyD88	Myeloid differentiation primary response protein 88
NF-κB	Nuclear factor kappa-light-chain-enhancer of activated B cells
NR	Not reported
Nrf2	Nuclear factor erythroid 2-related factor 2
NO	Nitric oxide
OS	Oxidative stress
PAMPs	Pathogen-associated molecular patterns
PGE_2_	Prostaglandin E_2_
P-gp	P-glycoprotein
RA	Rheumatoid arthritis
RANKL	Receptor activator of nuclear factor κB ligand
RCT	Randomized controlled trial
RNS	Reactive nitrogen species
ROS	Reactive oxygen species
RSE	*Rhodiola rosea* extract
SF	Saffron (*Crocus sativus* L.) extract
SHA	Stredná hyaluronan (medium molecular weight)
SOD	Superoxide dismutase
STAT	Signal transducer and activator of transcription
STAT3	Signal transducer and activator of transcription 3
TGF-β	Transforming growth factor-beta
Th1	T helper 1 cell
Th17	T helper 17 cell
TLR	Toll-like receptor
TNF	Tumor necrosis factor
TNF-α	Tumor necrosis factor-alpha
tsDMARDs	Targeted synthetic disease-modifying antirheumatic drugs
VHA	Very-high-molecular-weight hyaluronan
**↑**	Increase/elevate
**↓**	Decrease/suppress
